# Comprehensive Medication Review Completion Rates and Disparities After Medicare Star Rating Measure

**DOI:** 10.1001/jamahealthforum.2024.0807

**Published:** 2024-05-03

**Authors:** Anna Hung, Lauren Wilson, Valerie A. Smith, Juliessa M. Pavon, Caroline E. Sloan, Susan N. Hastings, Joel Farley, Matthew L. Maciejewski

**Affiliations:** 1 Department of Population Health Sciences, Duke University School of Medicine, Durham, North Carolina; 2 Duke-Margolis Center for Health Policy, Durham, North Carolina; 3 Center of Innovation to Accelerate Discovery and Practice Transformation, Durham VA Health Care System, Durham, North Carolina; 4 Division of General Internal Medicine, Department of Medicine, Duke University School of Medicine, Durham, North Carolina; 5 Division of Geriatrics, Department of Medicine, Duke University School of Medicine, Durham, North Carolina; 6 Center for the Study of Aging and Human Development, Duke University, Durham, North Carolina; 7 Geriatrics Research, Education, and Clinical Center, Durham Veterans Affairs Health Care System, Durham, North Carolina; 8 Department of Pharmaceutical Care & Health Systems, University of Minnesota College of Pharmacy, Minneapolis

## Abstract

**Importance:**

Comprehensive medication reviews (CMRs) are offered to qualifying US Medicare beneficiaries annually to optimize medication regimens and therapeutic outcomes. In 2016, Medicare adopted CMR completion as a Star Rating quality measure to encourage the use of CMRs.

**Objective:**

To examine trends in CMR completion rates before and after 2016 and whether racial, ethnic, and socioeconomic disparities in CMR completion changed.

**Design, Setting, and Participants:**

This observational study using interrupted time-series analysis examined 2013 to 2020 annual cohorts of community-dwelling Medicare beneficiaries aged 66 years and older eligible for a CMR as determined by Part D plans and by objective minimum eligibility criteria. Data analysis was conducted from September 2022 to February 2024.

**Exposure:**

Adoption of CMR completion as a Star Rating quality measure in 2016.

**Main Outcome and Measures:**

CMR completion modeled via generalized estimating equations.

**Results:**

The study included a total of 561 950 eligible beneficiaries, with 253 561 in the 2013 to 2015 cohort (median [IQR] age, 75.8 [70.7-82.1] years; 90 778 male [35.8%]; 6795 Asian [2.7%]; 24 425 Black [9.6%]; 7674 Hispanic [3.0%]; 208 621 White [82.3%]) and 308 389 in the 2016 to 2020 cohort (median [IQR] age, 75.1 [70.4-80.9] years; 126 730 male [41.1%]; 8922 Asian [2.9%]; 27 915 Black [9.1%]; 7635 Hispanic [2.5%]; 252 781 White [82.0%]). The unadjusted CMR completion rate increased from 10.2% (7379 of 72 225 individuals) in 2013 to 15.6% (14 185 of 90 847 individuals) in 2015 and increased further to 35.8% (18 376 of 51 386 individuals) in 2020, in part because the population deemed by Part D plans to be MTM-eligible decreased by nearly half after 2015 (90 487 individuals in 2015 to 51 386 individuals in 2020). Among a simulated cohort based on Medicare minimum eligibility thresholds, the unadjusted CMR completion rate increased but to a lesser extent, from 4.4% in 2013 to 12.6% in 2020. Compared with White beneficiaries, Asian and Hispanic beneficiaries experienced greater increases in likelihood of CMR completion after 2016 but remained less likely to complete a CMR. Dual-Medicaid enrollees also experienced greater increases in likelihood of CMR completion as compared with those without either designation, but still remained less likely to complete CMR.

**Conclusion and Relevance:**

This study found that adoption of CMR completion as a Star Rating quality measure was associated with higher CMR completion rates. The increase in CMR completion rates was achieved partly because Part D plans used stricter eligibility criteria to define eligible patients. Reductions in disparities for eligible Asian, Hispanic, and dual-Medicaid enrollees were seen, but not eliminated. These findings suggest that quality measures can inform plan behavior and could be used to help address disparities.

## Key Points

**Question:**

How did US nationwide comprehensive medication review (CMR) completion rates and disparities change after implementation of a 2016 Medicare Star Rating quality measure?

**Findings:**

This observational study of 561 950 Medicare beneficiaries using interrupted time-series analysis found that CMR completion rates increased in part due to plans reducing the size of the medication therapy management–eligible population by redefining their eligibility criteria. Asian, Hispanic, and low-income beneficiaries experienced greater increases in CMR completion rates, but rates remained lower than White beneficiaries and non–low-income beneficiaries.

**Meaning:**

Adoption of CMR completion as a Star Rating quality measure led to higher completion rates, and reductions in, but not elimination of, disparities for Asian, Hispanic, and low-income beneficiaries.

## Teaser

This cross-sectional study analyzes changes and disparities in comprehensive medication review (CMR) completion rates before and after implementation of a 2016 Medicare Star Rating quality measure.

## Introduction

Each year, millions of US Medicare beneficiaries nationwide are eligible for medication therapy management (MTM) services provided by Part D plans through the Medicare-mandated MTM program to optimize medication regimens and therapeutic outcomes.^1,2^ A core MTM service is the comprehensive medication review (CMR), in which a pharmacist or other qualified health care clinician initiates contact with a patient (often a telephone call, but it could also be face-to-face, or part of a telehealth visit), reviews all of the medications a patient is taking, identifies any medication-related problems, discusses with the patient, documents a written summary in the Medicare standardized format,^3^ and follows up with the prescriber as needed.^4,5^ CMRs have been shown to reduce medication-related problems, and in some cases, can lead to reductions in hospitalization and all-cause mortality.^4,6,7^

Since the inception of Medicare Part D in 2006, Medicare has required that Part D plans offer a CMR annually to beneficiaries who meet eligibility criteria. Medicare sets minimum eligibility thresholds as (1) having at least 2 to 3 chronic conditions, (2) taking at least 2 to 8 prescription drugs, and (3) having have high annual Part D drug spending (at least $4935 in 2023).^8,9^ Each Part D plan then sets their own MTM eligibility criteria based on these thresholds to determine to whom they offer a CMR. Prior studies^10,11^ have examined patient characteristics associated with offer and completion of a CMR in 2013 to 2016 as well as racial, ethnic, and socioeconomic disparities in Medicare minimum thresholds for MTM eligibility criteria.^12,13,14^ Specifically, Black and Hispanic beneficiaries were found to be less likely to meet Medicare minimum thresholds for MTM eligibility criteria,^12,13,14^ and low-income beneficiaries were less likely to be offered a CMR, as well as complete a CMR, if offered.^10,11^

CMRs have been underutilized historically, with only 17% of eligible Medicare beneficiaries receiving CMRs in 2014.^1^ To encourage utilization, Medicare added CMR completion as a Star Ratings measure starting in 2016, where CMR completion is defined as receipt of at least 1 CMR during the measurement year^15^ and requires documentation (with a written summary in Medicare standardized format). Individual Star Ratings measures contribute to the final summary star rating for each Part D plan that is shown on the Medicare Plan Finder website when beneficiaries are choosing Part D plans, are tied to financial incentives, and can influence beneficiary choice in plan (eAppendix in Supplement 1).^16^ To our knowledge, no studies have examined nationwide CMR completion rates following the 2016 adoption of CMR completion as a Star Ratings quality measure, and whether disparities have changed. To address this gap, this study assesses changes in CMR completion rates after the adoption of the 2016 Star Rating quality measure, and whether disparities in CMR completion changed across racial, ethnic, or socioeconomic group.

## Methods

### Study Population and Measures

This observational study using interrupted time-series analysis was approved by the institutional review board at Duke University Health System and followed the Strengthening the Reporting of Observational Studies in Epidemiology (STROBE) reporting guideline. A waiver of informed consent was approved by the Duke University Health System because this was secondary analysis that met the requirements of the Common Rule. The primary study cohort was derived from 2013 to 2020 Medicare 5% administrative claims data linked to 100% Part D MTM data. Patients consisted of community-dwelling Medicare Part D beneficiaries aged 66 years and older who met MTM eligibility criteria (as defined by each Part D plan) for at least 1 year in the study period and were continuously enrolled in Medicare Parts A, B, and D in the year prior to eligibility (eFigure 1 in Supplement 1). Identification of community-dwelling beneficiaries was based on excluding long-term care nursing home residents per a previously published Medicare claims-based algorithm.^17^ Beneficiaries were also excluded if they were enrolled in a plan that participated in the enhanced MTM model, which provided further flexibility in services and eligibility criteria and was being tested by Medicare between 2017 and 2021 in 5 regions by 6 plan sponsors.^18^ Similar to actual practice with the MTM program, beneficiaries could be represented in multiple years within the 2013 to 2020 study period if they were deemed eligible for MTM services in multiple years.

In additional analyses, we identified a secondary cohort by simulating an objective MTM-eligible population based on Medicare minimum eligibility criteria (ie, ≥3 common conditions, ≥8 Part D drugs, and $4255 in Part D drug spending for the year based on the 2020 threshold; see the eMethods in Supplement 1).^19^ Plans changed their eligibility criteria over time, so this cohort allowed us to also report CMR completion rates using a consistent and objective denominator for the entire study period.

The primary outcome in both cohorts was CMR completion, defined as receipt of at least 1 CMR in the measurement year as reported in the Medicare MTM data file, which is based on information submitted by Part D plan sponsors to the CMS Health Plan Management System.^20^ This definition is in accordance with the Star Rating quality measure.^15^ The patient characteristics of interest included age, sex, race and ethnicity (as ascertained from the Centers for Medicare & Medicaid Services [CMS] Common Medicare Environment and originally sourced from Social Security Administration data), dual-Medicaid enrollment or low-income subsidy (LIS; categorized as dual-Medicaid enrollment which automatically includes LIS, LIS-only, and neither dual-Medicaid enrollment nor LIS) in the year prior to date of eligibility, geographic region, rural residence,^21^ Charlson Comorbidity Index score, individual comorbidities, health care utilization, and medication utilization. Race and ethnicity categories included Asian, Black, Hispanic, North American Native, White, unknown, and other (defined as any other race or ethnicity not otherwise specified). Comorbidities (ie, diabetes, heart failure, hyperlipidemia, hypertension, chronic obstructive pulmonary disease, asthma, osteoporosis, depression, chronic kidney disease, dementia, and hearing loss) were identified based on Medicare Chronic Conditions Data Warehouse indicators in the year prior to the date of MTM eligibility,^22^ except for dementia and hearing loss, which were not available for the entire 2013 to 2020 time frame and were instead based on algorithms used in prior studies.^23,24^ Health care utilization was constructed as any inpatient stay, any emergency department visit, number of outpatient visits, and total costs to Medicare in the year prior to MTM eligibility. Medication utilization was constructed as the number of unique medications filled, number of prescription fills, out-of-pocket medication costs, number of unique potentially inappropriate medications (PIMs) filled, and number of PIM prescription fills measured in the year prior to MTM eligibility. PIMs were defined based on the Use of High-Risk Medications in the Elderly quality measure.^25^

### Statistical Analysis

Patient characteristics among those who met MTM eligibility criteria from 2013 to 2015 vs from 2016 to 2020, as well as those who completed vs those who did not complete CMR, were compared using standardized mean differences (SMDs). SMDs less than 10% indicate negligible difference between groups.^26^

Unadjusted annual CMR completion rates were reported from 2013 to 2020. In adjusted analyses applying an interrupted time series design to evaluate time trends and patient factors associated with CMR completion, models fit with generalized estimating equations with binomial error distributions and log links pooled yearly subcohorts with clustering by patient (because the same patient could be included in different yearly subcohorts). To determine whether there was a change in probability of CMR completion after introduction of the Star Rating quality measure, we modeled time continuously, allowing for a 1-time jump (ie, change in intercept) at 2016, corresponding to the introduction of the Star Rating quality measure, and a change in slope beginning at that time to allow differential trends to occur after that change. For easier interpretation, we reported annual adjusted risk ratios (aRRs) for the 2 slopes (2013-2015 and 2016-2020), and a 1-time aRR representing the 1-time incremental difference seen from 2015 to 2016 corresponding to the immediate change in policy (ie, change in intercept at 2016). In addition to these time terms, adjusted analyses included the aforementioned patient characteristics and included the Charlson Comorbidity Index score instead of individual comorbidities. In sensitivity analyses, the Charlson Comorbidity Index score was replaced by individual comorbidities and the time term parameter estimates were found to be robust (ie, not substantially change; data not shown).

To determine whether the Star Rating quality measure was associated with CMR completion differently across racial, ethnic, or socioeconomic groups, we ran 2 additional models that included interaction terms between all time terms and subgroup indicators (model 1, race and ethnicity; model 2, dual-Medicaid, LIS-alone, or neither). We compared the aRRs for the 2013 to 2015 slope, 2016 to 2020 slope, and change in intercept at 2016 between each subgroup and the reference group, and also compared the model-estimated probability that an individual in each subgroup completed CMR each year from 2013 to 2020 using tests of the asymptotic χ^2^ distribution of the likelihood ratio statistic. Model-estimated probabilities were calculated at the means values of all adjustment covariates. A statistically significant difference was defined at an α level of .05 or a 2-sided *P *value < .05. All analyses were conducted in SAS version 9.4 (SAS Institute). Data analysis was conducted from September 2022 to February 2024.

## Results

### Characteristics of Beneficiaries Deemed MTM-Eligible by Part D Plans

The study included a total of 561 950 MTM-eligible beneficiaries, with 253 561 in the 2013 to 2015 cohort (median [IQR] age, 75.8 [70.7-82.1] years; 90 778 male [35.8%]; 6795 Asian [2.7%]; 24 425 Black [9.6%]; 7674 Hispanic [3.0%]; 208 621 White [82.3%]) and 308 389 in the 2016 to 2020 cohort (median [IQR] age, 75.1 [70.4-80.9] years; 126 730 male [41.1%]; 8922 Asian [2.9%]; 27 915 Black [9.1%]; 7635 Hispanic [2.5%]; 252 781 White [82.0%]) (Table 1). The majority of participants across time frames had neither dual-Medicaid enrollment nor LIS (158 366 beneficiaries [62.5%] in 2013-2015 and 205 275 beneficiaries [66.6%] in 2016-2020). Over three-quarters had hyperlipidemia (190 257 [75.0%] in 2013-2015 and 242 223 [78.5%] in 2016-2020) and nearly one-half had a prior-year emergency department visit (120 488 [47.5%] in 2013-2015 and 152 876 [49.6%] in 2016-2020). Patients filled a median (IQR) of 15.0 (12.0-20.0) unique medications in 2013 to 2015 and 16.0 (13.0-20.0) unique medications in 2016 to 2020; a median (IQR) of 1.0 (0.0-1.0) PIMS in 2013 to 2015 and 0.0 (0.0-1.0) PIMs in 2016 to 2020; and paid a median (IQR) of $717.40 ($163.90-$1609.80) in 2013 to 2015 and $824.90 ($150.80-$1745.10) in 2016 to 2020 in annual out-of-pocket medication costs in the prior year (eTable 1 in Supplement 1).

**Table 1.** Characteristics of MTM-Eligible Beneficiaries and Completers vs Noncompleters of CMRs Before vs After 2016 Star Rating Measure

Characteristic	Before Star Rating quality measure (2013-2015), No. %			No CMR vs CMR, SMD, %	After Star Rating quality measure (2016-2020), No %			SMD, %	
								No CMR vs CMR	MTM-eligible before vs after quality measure
	Overall MTM-eligible (N = 253 561)	No CMR (n = 222 134)	CMR (n = 31 427)		Overall MTM-eligible (N = 308 389)	No CMR (n = 223 859)	CMR (n = 84 530)		
Age at date of MTM eligibility, median (IQR), y	75.8 (70.7-82.1)	75.9 (70.7-82.3)	75.3 (70.5-81.0)	9.9	75.1 (70.4-80.9)	75.1 (70.5-81.0)	74.9 (70.4-80.5)	5.5	9.2
Male sex	90 788 (35.8)	79 999 (36.0)	10 789 (34.3)	3.5	126 730 (41.1)	92 565 (41.3)	34 165 (40.4)	1.9	10.9
Race and ethnicity									
Asian	6795 (2.7)	6356 (2.9)	439 (1.4)	17.0	8922 (2.9)	6944 (3.1)	1978 (2.3)	8.7	10.2
Black	24 425 (9.6)	21 344 (9.6)	3081 (9.8)		27 915 (9.1)	20 295 (9.1)	7620 (9.0)		
Hispanic	7674 (3.0)	7221 (3.3)	453 (1.4)		7635 (2.5)	6033 (2.7)	1602 (1.9)		
North American Native	1151 (0.5)	1046 (0.5)	105 (0.3)		1681 (0.5)	1374 (0.6)	307 (0.4)		
White	208 621 (82.3)	181 704 (81.8)	26 917 (85.6)		252 781 (82.0)	182 142 (81.4)	70 639 (83.6)		
Other^a^	3463 (1.4)	3164 (1.4)	299 (1.0)		4857 (1.6)	3723 (1.7)	1134 (1.3)		
Unknown	1432 (0.6)	1299 (0.6)	133 (0.4)		4598 (1.5)	3348 (1.5)	1250 (1.5)		
Dual-Medicaid enrollment/LIS									
No dual-Medicaid enrollment and no LIS	158 366 (62.5)	136 833 (61.6)	21 533 (68.5)	15.3	205 275 (66.6)	147 455 (65.9)	57 820 (68.4)	6.1	8.6
LIS only	10 773 (4.2)	9419 (4.2)	1354 (4.3%)		11 177 (3.6)	7974 (3.6)	3203 (3.8)		
Dual-Medicaid enrollment	84 422 (33.3)	75 882 (34.2)	8540 (27.2)		91 937 (29.8)	68 430 (30.6)	23 507 (27.8)		
Geographic region									
Northeast	62 022 (24.5)	54 045 (24.3)	7977 (25.4)	9.2	72 024 (23.4)	52 297 (23.4)	19 727 (23.3)	4.7	8.9
Midwest	47 455 (18.7)	41 268 (18.6)	6187 (19.7)		63 554 (20.6)	45 447 (20.3)	18 107 (21.4)		
Other	274 (0.1)	242 (0.1)	32 (0.1)		399 (0.1)	326 (0.1)	73 (0.1)		
South	105 155 (41.5)	91 847 (41.3)	13 308 (42.3)		118 335 (38.4)	87 052 (38.9)	31 283 (37.0)		
West	38 655 (15.2)	34 732 (15.6)	3923 (12.5)		54 077 (17.5)	38 737 (17.3)	15 340 (18.1)		
Rural residence	68 198 (26.9)	58 787 (26.5)	9411 (29.9)	7.7	79 925 (25.9)	56 723 (25.3)	23 202 (27.4)	4.8	2.2
Charlson Comorbidity Index score, median (IQR)	1.0 (0.0-3.0)	1.0 (0.0-3.0)	1.0 (0.0-3.0)	10.5	2.0 (0.0-4.0)	2.0 (0.0-4.0)	1.0 (0.0-4.0)	11.8	10.8

Abbreviations: CMR, comprehensive medication review; LIS, low-income subsidy; MTM, medication therapy management; SMD, standardized mean difference.

^a^ Other race and ethnicity was defined as any other race and ethnicity not otherwise specified.

When comparing across time periods, beneficiaries deemed MTM-eligible by Part D plans in 2016 to 2020 (vs 2013-2015) were more likely to be male (126 730 beneficiaries [41.1%] vs 90 788 beneficiaries [35.8%]; SMD, 10.9%), have a higher Charlson Comorbidity Index score (median [IQR] of 2.0 [0.0-4.0] vs 1.0 [0.0-3.0]; SMD; 10.8%), have diagnosed hearing loss (14 023 beneficiaries [4.5%] vs 5427 [2.1%], SMD, 13.4%), and use fewer PIMs (median [IQR] of 0.0 [0.0-1.0] vs 1.0 [0.0-1.0]; SMD, 17.1%), and have fewer fills of those PIMs (median [IQR] of 0.0 [0.0-4.0] vs 1.0 [0.0-5.0]; SMD, 17.6%) (Table 1 and eTable 1 in Supplement 1). There were also slight decreases in the proportion of Hispanic beneficiaries (7674 beneficiaries [3.0%] to 7635 beneficiaries [2.5%]; overall SMD, 10.2%), dual-Medicaid beneficiaries (84 422 beneficiaries [33.3%] to 91 937 beneficiaries [29.8%]; overall SMD, 8.6%), and LIS-only beneficiaries (10 773 beneficiaries [4.2%] to 11 177 beneficiaries [3.6%]; overall SMD, 8.6%) who were deemed MTM-eligible. Similar trends were seen for the second (simulated) study cohort except that they had a higher Charlson Comorbidity Index score (median [IQR] of 4.0 [2.0-6.0] 2013-2015 and 4.0 [3.0-6.0] in 2016-2020 in the simulated cohort versus 1.0 [0.0-3.0] in 2013-2015 and 2.0 [0.0-4.0] in the primary cohort) (Table 1 and eTable 2 in Supplement 1). Prior to 2016, the simulated cohort was also less likely to be dual-Medicaid or LIS-only beneficiaries (107 441 beneficiaries [47.7%] with neither designation in simulated cohort versus 158 366 [62.5%] in primary cohort) (Table 1 and eTable 2 in Supplement 1).

### Characteristics of Beneficiaries Completing CMRs

In adjusted analyses (which also adjusted for time trends), patient characteristics associated with lower likelihood of completing CMR among the population deemed MTM-eligible by plans from 2013 to 2020 included older age; male sex; Asian, Hispanic, North American Native, other, and unknown race and ethnicity (as compared with White race); dual-Medicaid enrollment (as compared with no dual-Medicaid enrollment or LIS); all regions (as compared with the Northeast); and greater number of comorbidities (eTable 3 in Supplement). Patient characteristics associated with increased likelihood of completing CMR included rural residence and use of a greater number of medications in the prior year.

### CMR Completion Before and After 2016

**Figure 1.**
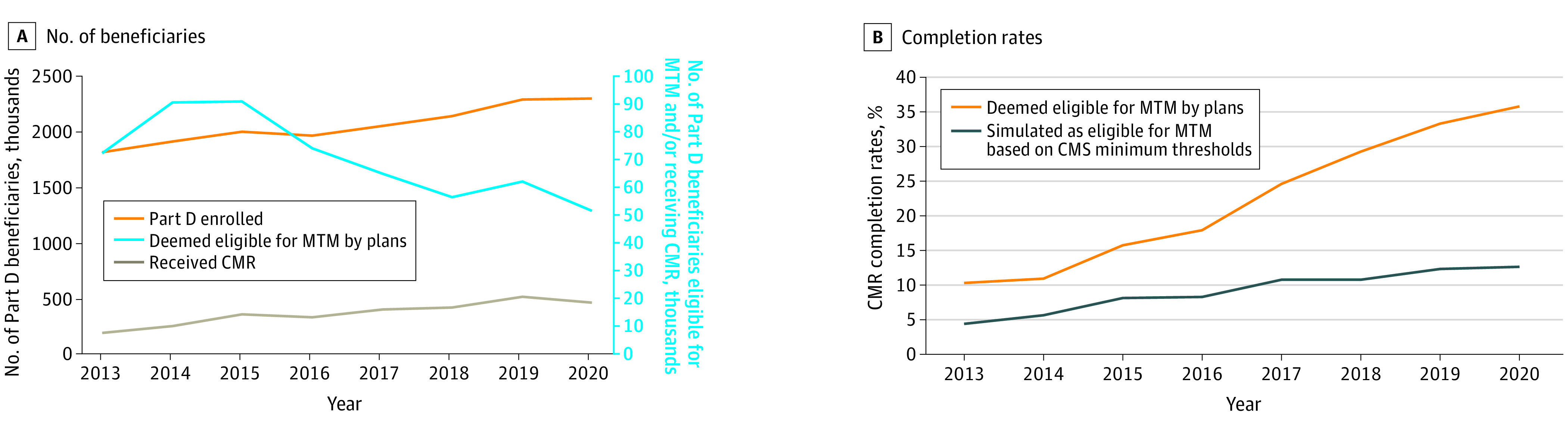
Unadjusted Number of Eligible Part D Beneficiaries and Medication Therapy Management (MTM) Completion Rates, 2013-2020 The figure shows the unadjusted number of Medicare Part D beneficiaries who were MTM eligible (A) and the unadjusted MTM comprehensive medication review (CMR) completion rates (B). CMS indicates the Centers for Medicare & Medicaid Services.

From 2013 to 2020, the number of CMR recipients increased from 7379 to 18 376 (Figure 1A). While the number of beneficiaries deemed MTM-eligible by plans increased from 2013 to 2015, this number decreased from 90 847 in 2015 to 51 386 in 2020 (Figure 1A). As a result, among the population deemed MTM-eligible by plans, the unadjusted CMR completion rate increased from 10.2% in 2013 (7379 of 72 225) to 15.6% in 2015 (14 185 of 90 847) and then to 35.8% (18 376 of 51 386 individuals) in 2020 (Figure 1B), representing an increase of 3.5 times across the entire study period. Among a simulated MTM-eligible population based on applying CMS minimum eligibility thresholds consistently across years (ie, no changes in the eligibility criteria from year to year such as was evident by plans), the unadjusted CMR completion rate increased as well, but to a lesser extent, from 4.4% to 8.1% from 2013 to 2015 and then to 12.6% in 2020 (Figure 1B), representing an increase of 2.9 times across the entire study period. The size of the simulated MTM-eligible population increased year over year from 2013 to 2020 (eTable 4 in Supplement 1).

In adjusted analyses (Table 2), among the population deemed MTM-eligible by plans, CMR completion rates increased each year prior to the Star Rating measure (aRR each year compared with the prior year, 1.30; 95% CI, 1.28-1.31) but moderated slightly (aRR each year, 1.26; 95% CI, 1.22-1.30) after a 1-time increase in 2016 (aRR, 1.07; 95% CI, 1.04-1.09). Similar results were seen for the simulated MTM-eligible population (based on Medicare minimum thresholds), but with a much flatter slope after 2016 (aRR each year, 1.13; 95% CI, 1.08-1.19) (eTable 5 in Supplement 1).

**Table 2.** Change in Medication Therapy Management Comprehensive Medication Review Completion Rates Before vs After 2016 Star Rating Measure (N = 561 950)

Model^a^	Adjusted risk ratio (95% CI)	*P* value^b^
**Model 1 (averaged over all populations)**		
Slope 2013-2015	1.30 (1.28-1.31)	NA
Change in intercept at 2016	1.07 (1.04-1.09)	NA
Slope 2016-2020	1.26 (1.22-1.30)	NA
**Model 2 (by race and ethnicity)**		
Asian beneficiaries		
Slope 2013-2015	1.54 (1.33-1.78)	.006
Change in intercept at 2016	1.16 (0.95-1.41)	.36
Slope 2016-2020	1.33 (0.99-1.80)	.001
Black beneficiaries		
Slope 2013-2015	1.28 (1.19-1.37)	.67
Change in intercept at 2016	0.98 (0.88-1.09)	.03
Slope 2016-2020	1.30 (1.12-1.50)	.001
Hispanic beneficiaries		
Slope 2013-2015	1.31 (1.13-1.51)	.86
Change in intercept at 2016	1.38 (1.11-1.70)	.006
Slope 2016-2020	1.32 (0.99-1.77)	.007
North American Native beneficiaries		
Slope 2013-2015	1.12 (0.86-1.47)	.28
Change in intercept at 2016	1.02 (0.66-1.59)	.82
Slope 2016-2020	1.26 (0.71-2.23)	.95
White beneficiaries (reference group)		
Slope 2013-2015	1.29 (1.27-1.31)	NA
Change in intercept at 2016	1.07 (1.04-1.10)	NA
Slope 2016-2020	1.25 (1.21-1.30)	NA
Unknown race		
Slope 2013-2015	1.36 (1.05-1.75)	.68
Change in intercept at 2016	1.14 (0.85-1.52)	.64
Slope 2016-2020	1.33 (0.80-2.24)	.008
Other race^c^		
Slope 2013-2015	1.31 (1.11-1.54)	.86
Change in intercept at 2016	1.28 (1.01-1.64)	.01
Slope 2016-2020	1.25 (0.89-1.75)	.85
**Model 3 (by socioeconomic subgroup)**		
Neither dual-Medicaid nor LIS enrollee (reference group)		
Slope 2013-2015	1.32 (1.30-1.35)	NA
Change in intercept at 2016	1.08 (1.05-1.11)	NA
Slope 2016-2020	1.23 (1.18-1.28)	NA
Dual-Medicaid enrollee		
Slope 2013-2015	1.24 (1.18-1.31)	<.001
Change in intercept at 2016	1.03 (0.95-1.12)	.14
Slope 2016-2020	1.35 (1.21-1.50)	<.001
LIS-only enrollee		
Slope 2013-2015	1.21 (1.10-1.32)	.01
Change in intercept at 2016	1.12 (0.96-1.31)	.50
Slope 2016-2020	1.27 (1.05-1.54)	.02

Abbreviations: LIS, low-income subsidy; NA, not applicable.

^a^ All 3 models were adjusted for age, sex, race and ethnicity, dual-Medicaid enrollment and low-income subsidy, region, rural residence, Charlson Comorbidity Index score, and prior-year utilization (eg, had inpatient stay, had emergency department visit, number of outpatient visits, inflation-adjusted costs, number of unique medications, number prescription fills, inflation-adjusted out-of-pocket medication costs, and number of potentially inappropriate medications). Models 2 and 3 included interaction terms among each of the time terms and each of the racial and ethnic and socioeconomic subgroups, respectively.

^b^ Test between subgroup and reference group for a given time term.

^c^ Other race and ethnicity was defined as any other race and ethnicity not otherwise specified.

### Reduction in Racial and Ethnic Disparities in CMR Completion Rates After 2016

**Figure 2.**
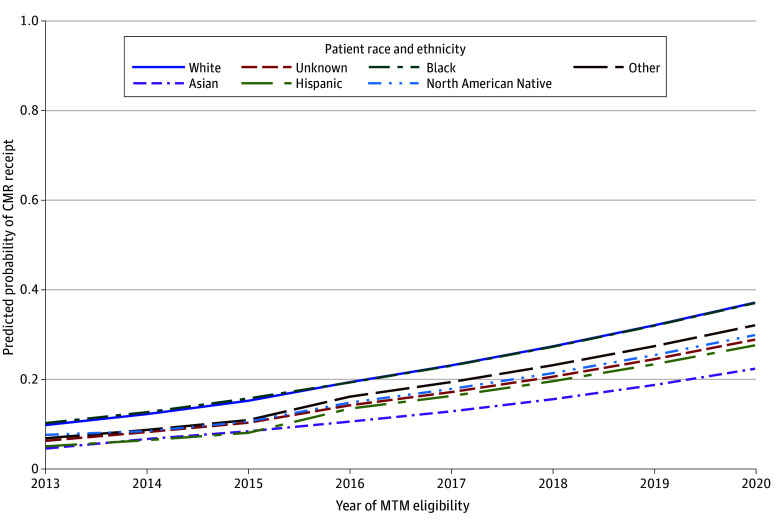
Model-Based Probabilities of Medication Therapy Management (MTM) Comprehensive Medication Review (CMR) Completion by Racial or Ethnic Group Model 2 adjusted for age, sex, race and ethnicity, dual-Medicaid enrollment or low-income subsidy, region, rural residence, Charlson Comorbidity Index score, and prior-year utilization (eg, had inpatient stay, had emergency department visit, number of outpatient visits, inflation-adjusted costs, number of unique medications, number prescription fills, inflation-adjusted out-of-pocket medication costs, and number of potentially inappropriate medications), and included interaction terms between each of the time terms and the race and ethnicity indicator. Other race and ethnicity was defined as any other race and ethnicity not otherwise specified.

Compared with White beneficiaries, Hispanic beneficiaries had a greater 1-time jump at 2016 (aRR, 1.38; 95% CI, 1.11-1.70 vs aRR, 1.07; 95% CI, 1.04-1.10; *P* = .006) and slope after 2016 (aRR, 1.32; 95% CI, 0.99-1.77 vs aRR, 1.25; 95% CI, 1.21-1.30; *P* = .007) and Asian beneficiaries had greater slopes before 2016 (aRR = 1.54; 95% CI, 1.33-1.78 vs aRR, 1.29, 95% CI, 1.27-1.31; *P* = .006) and after 2016 (aRR, 1.33; 95% CI, 0.99-1.80 vs aRR, 1.25; 95% CI, 1.21-1.30; *P* = .001) (Table 2). However, Asian and Hispanic beneficiaries continued to have lower estimated year-to-year CMR completion rates compared with White beneficiaries both before and after 2016 (Figure 2 and eTable 6 in Supplement 1).

Compared with White beneficiaries, Black beneficiaries had a smaller 1-time jump at 2016 but a greater post-2016 slope (Table 2), and model-estimated probabilities did not differ between Black beneficiaries and White beneficiaries (Figure 2 and eTable 6 in Supplement 1). Similar results across race and ethnicity were seen in the simulated MTM-eligible cohort (eTable 5 in Supplement 1).

### Reduction in Socioeconomic Disparities in CMR Completion Rates After 2016

**Figure 3.**
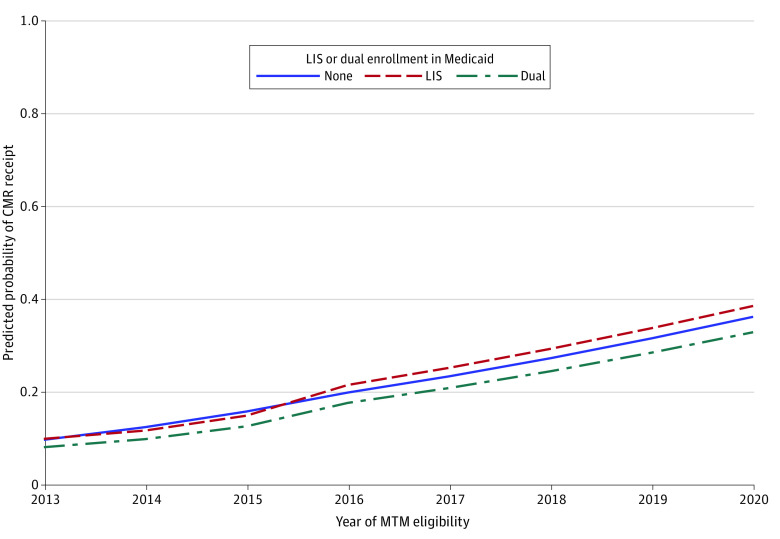
Model-based probabilities of Medication Therapy Management (MTM) Comprehensive Medication Review (CMR) Completion by Dual-Medicaid or Low-Income Subsidy (LIS) Group Model 3 adjusted for age, sex, race and ethnicity, dual-Medicaid enrollment or low-income subsidy, region, rural residence, Charlson Comorbidity Index score, and prior-year utilization (eg, had inpatient stay, had emergency department visit, number of outpatient visits, inflation-adjusted costs, number of unique medications, number prescription fills, inflation-adjusted out-of-pocket medication costs, and number of potentially inappropriate medications), and included interaction terms between each of the time terms and the dual-Medicaid or LIS-only indicator.

Compared with those without Medicaid or LIS, dual-Medicaid enrollees and LIS-only enrollees both had smaller pre-2016 slopes (aRR for neither Medicaid nor LIS, 1.32; 95% CI, 1.30-1.35; aRR for dual-Medicaid, 1.24; 95% CI, 1.18-1.31; *P* < .001; aRR for LIS-only, 1.21; 95% CI, 1.10-1.32; *P* = .01) and greater post-2016 slopes (aRR for neither, 1.23; 95% CI, 1.18-1.28; aRR for dual-Medicaid, 1.35; 95% CI, 1.21-1.50; *P* < .001; aRR for LIS only, 1.27; 95% CI, 1.05-1.54; *P* = .02) (Table 2). Similar results by socioeconomic status were seen in the simulated MTM-eligible cohort pertaining to the post-2016 slopes, with differences pertaining to the pre-2016 slopes and intercepts (eTable 5 in Supplement 1). Model-estimated probabilities for CMR completion were still lower for dual-Medicaid enrollees and similar for LIS-only enrollees when compared with those with neither Medicaid nor LIS both before and after 2016 (Figure 3 and eTable 6 in Supplement 1).

## Discussion

This observational study using interrupted time-series analysis found that following the adoption of CMR completion as a Star Rating measure in 2016, CMR completion increased from 10.2% in 2013 to 35.8% in 2020. However, this CMR completion increase was in part due to Part D plans dramatically decreasing the size of the population they deemed to be MTM-eligible. After 2016, Part D plans were more likely to use the most limiting eligibility thresholds set by Medicare (eg, the proportion of plans requiring beneficiaries to have at least 3 chronic conditions increased from 80% in 2016 to 90% in 2019 and the proportion of plans requiring beneficiaries to have at least 8 medications increased from 57% in 2016 to 78% in 2019).^2,27,28,29^ When we simulated a cohort using minimum eligibility thresholds, CMR completion increased to a lesser extent, from 4.4% in 2013 to 12.6% in 2020. This finding suggests that the adoption of CMR completion as a Star Rating measure created strong incentives for Part D plans to take actions that could impact the rates, which they appeared to act on by shrinking the denominator (eligible population) instead of focusing only on growing the numerator (number of CMRs provided).

Likely in part to address this shrinkage in the MTM-eligible population, Medicare proposed changes in December 2022 to their minimum thresholds for MTM eligibility criteria. These changes included lowering the Part D drug cost threshold from $4935 in 2023 to be commensurate with the average annual cost of 5 generic drugs ($1004 in 2020), lowering the minimum number of covered Part D drugs required from a range of 2 to 8 drugs to 2 to 5 drugs, and requiring that Medicare Part D plans target 10 specified core chronic diseases.^30^ The goal of these changes is “to promote consistent, equitable, and expanded access”^30^ to MTM. On April 5, 2024, CMS issued a final ruling on the proposed changes in MTM eligibility criteria that will take effect starting in 2025.^31^ The Part D drug cost threshold was lowered to $1623 (and is based on the average annual cost of eight generic drugs), and Part D plans must target all 10 core chronic diseases.^31^ The number of Part D drugs was not lowered, but may be lowered in the future. Given these changes, the estimated expansion in beneficiaries eligible for MTM is from approximately 3.6 million (7%) to 7.1 million (13%).^31^

Despite increases, CMR completion rates remained relatively low from 2013 to 2020, suggesting room for improvement and the need for additional strategies to help increase uptake. Even though the MTM program has been around since 2006, a key barrier to CMR completion is patient and clinician lack of awareness of the MTM program and the benefits of a CMR.^32,33^ Patient- and clinician-centered materials that educate on the value of CMRs, introduce the pharmacist who will conduct the CMR, and clarify that the CMR is covered by the Part D plan (ie, of no charge to the patient) could help increase CMR completion rates. Because clinicians are often overburdened, reporting time pressure and even greater burnout after the COVID-19 pandemic, a CMR that is already being conducted by a third-party pharmacist may provide valuable information for clinicians and represent an efficient use of limited resources.^34,35,36^ Another reported barrier is the lack of a relationship between the clinician and the MTM practitioner (who is typically a pharmacist) performing the CMR, because this MTM practitioner generally works for a community pharmacy, MTM vendor, or the Part D plan, and not directly in the clinician’s clinic.^37^ To address this barrier, novel approaches are needed. Perhaps one idea would be to contract MTM services to pharmacists who already work within a clinic and have established relationships with clinicians. When this is not feasible, another approach includes grouping MTM-eligible beneficiaries with the same prescriber to be assigned to the same MTM practitioner, thus encouraging the development of relationships over time. Further research is needed to explore ways to overcome barriers at the level of the patient, MTM practitioner, clinician, health system, Part D plan, pharmacy, MTM vendor, and the Medicare program.

We also examined whether the Star Rating measure was associated with CMR completion rate disparities by race, ethnicity, and socioeconomic status, given that prior studies have found that Black and Hispanic beneficiaries are less likely to meet Medicare minimum thresholds for MTM eligibility criteria^12,13,14^ and that among those offered a CMR, Black beneficiaries do not differ in their likelihood of CMR completion but other racial and ethnic minority groups are less likely to complete a CMR.^10^ Consistent with this prior work,^10,12,13,14^ we found that Black beneficiaries did not differ in likelihood of completing a CMR compared with White beneficiaries, but that Asian and Hispanic beneficiaries were less likely to complete a CMR than White beneficiaries. Asian and Hispanic beneficiaries’ completion rates did increase more than White beneficiaries over time, but overall remained lower, indicating that disparities were not eliminated and suggesting the need for additional strategies. Prior studies^10,11^ have also found that low-income beneficiaries were less likely to be offered a CMR, as well as complete a CMR if offered. Aligned with these prior findings, we saw that dual-Medicaid enrollees were less likely to receive a CMR and that prior to the Star Rating measure in 2016, dual-Medicaid enrollees and LIS-only enrollees had slower CMR completion growth rates. However, after 2016, dual-Medicaid enrollees and LIS-only enrollees’ CMR completion rates increased faster than that of non–low-income beneficiaries. Nonetheless, dual-Medicaid enrollees had lower CMR completion rates compared with non–low-income beneficiaries both before and after 2016, indicating that although this disparity was reduced, it was not completely resolved. Medicare’s focus on health equity, such as through new health equity quality measures and requirements to provide materials in non-English languages, may help to further reduce these disparities.^30^

While the present study focused on CMR completion rates, it is important to note that this type of quality measure is limited in that it is a process measure.^38^ New initiatives by measure development organizations are underway to develop the next generation of CMR-related quality measures, such as outcome measures that focus on what goal was achieved from the CMR.^39^ Furthermore, CMR completion rate is only 1 of 13 to 18 Star Rating measures depending on the calendar year (as described in the eAppendix in Supplement 1), so is only 1 consideration for Part D plans.

### Limitations and Strengths

There are several study limitations we must report. First, there was no comparator group because CMR completion was adopted as a Star Rating measure for all Medicare Part D plans. Additionally, there could be residual unobserved confounding due to the study being observational in design and there being unobserved factors, such as patient activation. Second, information on race and ethnicity was derived from Medicare data, which is more likely to be inaccurate as compared with self-reported data^40^; however, information on race and ethnicity was not available from self-reported data (eg, beneficiary surveys) for the entire study population. Third, we excluded beneficiaries (<11 per eFigure 1 in Supplement 1) enrolled in plans involved in a Medicare model testing Enhanced MTM, so the results are only generalizable to CMRs provided through traditional MTM programs (which represent the vast majority). A key strength of this study is examination of the long-term 2013 to 2020 trends in CMR completion.

## Conclusions

In conclusion, this observational study using interrupted time-series analysis found that the adoption of CMR completion rate as a Star Rating quality measure was associated with higher CMR completion rates, in part because Part D plans used stricter eligibility criteria to define eligible patients. Disparities in CMR completion rates were reduced for Asian, Hispanic, and dual-Medicaid enrollees, but not eliminated. These findings suggest that quality measures can inform plan behavior and could be used to help address disparities.

## Notes

Supplement 1. **eAppendix.** Additional Background on Star Rating Measures **eFigure 1.** Cohort Inclusion Diagram **eMethods.** Simulated Cohort Definition Based on Medicare’s Minimum Eligibility Thresholds **eTable 1.** Additional Characteristics of MTM-Eligible Beneficiaries and Completers vs Noncompleters of CMRs Before vs After 2016 Star Rating Measure **eTable 2.** Characteristics of MTM-Eligible Beneficiaries and Users vs Nonusers of CMRs Before vs After 2016 Star Rating Quality Measure (Using the Simulated MTM-Eligible Cohort Based on Medicare’s Minimum Eligibility Thresholds) **eTable 3.** Patient Characteristics Associated With MTM CMR Completion After Adjusting for Time Trends (n=561,950) **eTable 4.** Descriptive Trends of Cohort Simulated as Eligible for MTM Based on CMS Minimum Thresholds and Breakdown of Cohort Based on Inclusion Criteria **eTable 5.** Change in MTM CMR Completion Rates Before vs After 2016 Star Rating Measure Using the Simulated MTM-Eligible Cohort Based on Medicare’s Minimum Eligibility Thresholds (n=663,315) **eTable 6.** Model-based Estimated Probability of MTM CMR Completion by Subgroup and Year

Supplement 2.
Data Sharing Statement

## References

[1] Pestka DL, Zillich AJ, Coe AB, . Nationwide estimates of medication therapy management delivery under the Medicare prescription drug benefit. J Am Pharm Assoc (2003). 2020;60(3):456-461. doi:10.1016/j.japh.2019.12.002 31926872 PMC10478169

[2] Centers for Medicare and Medicaid Services. 2019 Medicare part d medication therapy management (MTM) programs: fact sheet summary of 2019 MTM programs. September 25, 2019. Accessed March 27, 2024. https://www.cms.gov/Medicare/Prescription-Drug-Coverage/PrescriptionDrugCovContra/Downloads/CY2019-MTM-Fact-Sheet.pdf

[3] Centers for Medicare and Medicaid Services. technical instructions for the standardized format for part D medication therapy management (MTM) program comprehensive medication review (CMR) summary. January 1, 2022. Updated February 29, 2024. Accessed March 27, 2024. https://www.cms.gov/medicare/coverage/prescription-drug-coverage-contracting/medication-therapy-management

[4] Burns A; American Pharmacists Association; National Association of Chain Drug Stores Foundation. Medication therapy management in pharmacy practice: core elements of an MTM service model (version 2.0). J Am Pharm Assoc (2003). 2008;48(3):341-353. doi:10.1331/JAPhA.2008.08514 18595820

[5] Agency for Healthcare Research and Quality. Improving medication safety in high risk Medicare beneficiaries toolkit. website. July 11, 2012. Accessed March 27, 2024. https://effectivehealthcare.ahrq.gov/products/medication-therapy-management-1/research

[6] Viswanathan M, Kahwati LC, Golin CE, . Medication therapy management interventions in outpatient settings: a systematic review and meta-analysis. JAMA Intern Med. 2015;175(1):76-87. doi:10.1001/jamainternmed.2014.5841 25401788

[7] Bloomfield HE, Greer N, Linsky AM, . Deprescribing for community-dwelling older adults: a systematic review and meta-analysis. J Gen Intern Med. 2020;35(11):3323-3332. doi:10.1007/s11606-020-06089-2 32820421 PMC7661661

[8] Centers for Medicare and Medicaid Services. Contract year 2022 part D medication therapy management program guidance and submission instructions. April 30, 2021. Accessed March 27, 2024. https://www.cms.gov/files/document/memo-contract-year-2022-medication-therapy-management-mtm-program-submission-v-043021.pdf

[9] Centers for Medicare and Medicaid Services. Contract year 2023 medication therapy management program information and submission instructions. April 15, 2022. Accessed March 27, 2024. https://www.cms.gov/files/document/memo-contract-year-2023-medication-therapy-management-mtm-program-submission-v041522.pdf

[10] Coe AB, Adeoye-Olatunde OA, Pestka DL, . Patterns and predictors of older adult Medicare Part D beneficiaries’ receipt of medication therapy management. Res Social Adm Pharm. 2020;16(9):1208-1214. doi:10.1016/j.sapharm.2019.12.007 31859225 PMC9827433

[11] Lee M, Zarowitz BJ, Pellegrin K, Cooke CE, Fleming SP, Brandt N. Social determinants predict whether Medicare beneficiaries are offered a comprehensive medication review. Res Social Adm Pharm. 2023;19(1):184-188. doi:10.1016/j.sapharm.2022.09.015 36216754

[12] Spivey CA, Wang J, Qiao Y, . Racial and ethnic disparities in meeting MTM eligibility criteria based on star ratings compared with the Medicare Modernization Act. J Manag Care Spec Pharm. 2018;24(2):97-107. doi:10.18553/jmcp.2018.24.2.97 29384031 PMC5793919

[13] Spivey CA, Qiao Y, Wang J, . Comparative effectiveness of medication therapy management eligibility criteria across racial/ethnic groups. J Am Geriatr Soc. 2019;67(3):581-587. doi:10.1111/jgs.15754 30674080 PMC6438366

[14] Wang J, Qiao Y. Historical trend of disparity implications of Medicare MTM eligibility criteria. Res Social Adm Pharm. 2013;9(6):758-769. doi:10.1016/j.sapharm.2012.09.003 23062785 PMC3549304

[15] Pharmacy Quality Alliance. 2022 PQA measure manual: completion rate for comprehensive medication review (CMR). 2022. Accessed October 1, 2022. https://www.pqaalliance.org/pqa-measures

[16] Reid RO, Deb P, Howell BL, Shrank WH. Association between Medicare Advantage plan star ratings and enrollment. JAMA. 2013;309(3):267-274. doi:10.1001/jama.2012.173925 23321765

[17] Goodwin JS, Li S, Zhou J, Graham JE, Karmarkar A, Ottenbacher K. Comparison of methods to identify long term care nursing home residence with administrative data. BMC Health Serv Res. 2017;17(1):376. doi:10.1186/s12913-017-2318-9 28558756 PMC5450097

[18] Centers for Medicare and Medicaid Services. Part D enhanced medication therapy management model. Updated April 26, 2022. https://innovation.cms.gov/innovation-models/enhancedmtm

[19] Centers for Medicare and Medicaid Services. CY 2020 medication therapy management program guidance and submission instructions. April 5, 2019. Accessed March 27, 2024. https://www.cms.gov/Medicare/Prescription-Drug-Coverage/PrescriptionDrugCovContra/Downloads/Memo-Contract-Year-2020-Medication-Therapy-Management-MTM-Program-Submission-v-041019-.pdf

[20] Research Data Assistance Center. Part D medication therapy management data file. August 17, 2020. Accessed March 27, 2024. https://resdac.org/cms-data/files/part-d-mtm

[21] Rural-Urban Commuting Area Codes. US Department of Agriculture Economic Research Service. Aug 17, 2020. Available from: https://www.ers.usda.gov/data-products/rural-urban-commuting-area-codes/

[22] Chronic Conditions. Chronic Conditions Data Warehouse. Available from: https://www2.ccwdata.org/web/guest/condition-categories-chronic.

[23] Grodstein F, Chang CH, Capuano AW, . Identification of Dementia in Recent Medicare Claims Data, Compared With Rigorous Clinical Assessments. J Gerontol A Biol Sci Med Sci. 2022;77(6):1272-1278. doi:10.1093/gerona/glab377 34919685 PMC9159666

[24] Deal JA, Reed NS, Kravetz AD, . Incident Hearing Loss and Comorbidity: A Longitudinal Administrative Claims Study. JAMA Otolaryngol Head Neck Surg. 2019;145(1):36-43. doi:10.1001/jamaoto.2018.2876 30419134 PMC6439817

[25] Pharmacy Quality Alliance. 2022 PQA Measure Manual: Use of High-Risk Medications in the Elderly (HRM). 2022. Accessed October 1, 2022. https://www.pqaalliance.org/

[26] Austin PC. Balance diagnostics for comparing the distribution of baseline covariates between treatment groups in propensity-score matched samples. Stat Med. 2009;28(25):3083-3107. doi:10.1002/sim.3697 19757444 PMC3472075

[27] Centers for Medicare and Medicaid Services. 2016 Medicare part D MEDICATION THERAPY MANAGEMENT (MTM) programs: fact sheet summary of 2016 MTM programs. May 4, 2016. Accessed March 27, 2024. https://www.cms.gov/Medicare/Prescription-Drug-Coverage/PrescriptionDrugCovContra/Downloads/CY2016-MTM-Fact-Sheet.pdf

[28] Centers for Medicare and Medicaid Services. 2017 Medicare part D medication therapy management (MTM) programs: fact sheet summary of 2017 MTM programs. August 16, 2017. Accessed March 24, 2024. https://www.cms.gov/Medicare/Prescription-Drug-Coverage/PrescriptionDrugCovContra/Downloads/CY2017-MTM-Fact-sheet.pdf

[29] Centers for Medicare and Medicaid Services. 2018 Medicare part D medication therapy management (MTM) programs: fact sheet summary of 2018 MTM programs. August 20, 2018. Accessed March 24, 2024. https://www.cms.gov/Medicare/Prescription-Drug-Coverage/PrescriptionDrugCovContra/Downloads/CY2018-MTM-Fact-Sheet.pdf

[30] Federal Register. Medicare program; contract year 2024 policy and technical changes to the Medicare advantage program, Medicare prescription drug benefit program, Medicare cost plan program, and programs of all-inclusive care for the elderly. April 12, 2023. Accessed March 27, 2024. https://www.federalregister.gov/documents/2023/04/12/2023-07115/medicare-program-contract-year-2024-policy-and-technical-changes-to-the-medicare-advantage-program

[31] Federal Register. Medicare Program; changes to the medicare advantage and the medicare prescription drug benefit program for contract year 2024--remaining provisions and contract year 2025 policy and technical changes to the Medicare Advantage Program, Medicare Prescription Drug Benefit Program, Medicare Cost Plan Program, and Programs of All-Inclusive Care for the Elderly (PACE). April 23, 2024. Accessed April 10, 2024. https://public-inspection.federalregister.gov/2024-07105.pdf

[32] Coe AB, Farris KB, Solway E, . Predictors of receipt of comprehensive medication reviews in older adults. J Gerontol A Biol Sci Med Sci. 2023;78(3):463-469. doi:10.1093/gerona/glac096 35446953 PMC9977218

[33] Taylor AM, Axon DR, Campbell P, . What patients know about services to help manage chronic diseases and medications: findings from focus groups on medication therapy management. J Manag Care Spec Pharm. 2018;24(9):904-910. doi:10.18553/jmcp.2018.24.9.904 30156456 PMC10398267

[34] Yates SW. Physician stress and burnout. Am J Med. 2020;133(2):160-164. doi:10.1016/j.amjmed.2019.08.034 31520624

[35] Porter J, Boyd C, Skandari MR, Laiteerapong N. Revisiting the Time Needed to Provide Adult Primary Care. J Gen Intern Med. 2023;38(1):147-155. doi:10.1007/s11606-022-07707-x 35776372 PMC9848034

[36] Gunja MZ, Gumas ED, Williams RD II, Doty MM, Shah A, Fields K. Stressed out and burned out: the global primary care crisis: findings from the 2022 international health policy survey of primary care physicians. The Commonwealth Fund. November 17, 2022. Accessed March 27, 2024. https://www.commonwealthfund.org/publications/issue-briefs/2022/nov/stressed-out-burned-out-2022-international-survey-primary-care-physicians

[37] McGrath SH, Snyder ME, Dueñas GG, Pringle JL, Smith RB, McGivney MS. Physician perceptions of pharmacist-provided medication therapy management: qualitative analysis. J Am Pharm Assoc (2003). 2010;50(1):67-71. doi:10.1331/JAPhA.2010.08186 20097641

[38] Donabedian A. The quality of care. How can it be assessed? JAMA. 1988;260(12):1743-1748. doi:10.1001/jama.1988.03410120089033 3045356

[39] Cost M. Aiming for the next generation of MTM Quality measures. Pharmacy Quality Alliance. July 28, 2023. Accessed March 27, 2024. https://pqa.memberclicks.net/index.php?option=com_dailyplanetblog&view=entry&year=2023&month=07&day=28&id=257:aiming-for-the-next-generation-of-mtm-quality-measures

[40] US Department of Health and Human Services Office of Inspector General. Inaccuracies in Medicare’s race and ethnicity data hinder the ability to assess health disparities. June 2022. Accessed March 27, 2024. https://oig.hhs.gov/oei/reports/OEI-02-21-00100.pdf

