# Error in Figure 2

**DOI:** 10.1001/jamahealthforum.2024.1508

**Published:** 2024-06-07

In the Original Investigation titled “Comprehensive Medication Review Completion Rates and Disparities After Medicare Star Rating Measure,”^1^ published May 3, 2024, there was an error in the legend of Figure 2, in which the race and ethnicity categories were mislabeled. This article has been corrected.^1^
