# Supplementary material for: Comprehensive Medication Review Completion Rates and Disparities After Medicare Star Rating Measure
Source: JAMA Health Forum. 2024 May 3;5(5):e240807. doi: 10.1001/jamahealthforum.2024.0807 (PMC11069085; doi:10.1001/jamahealthforum.2024.0807)
Supplement: Supplement 1. — eAppendix. Additional Background on Star Rating Measures eFigure 1. Cohort Inclusion Diagram eMethods. Simulated Cohort Definition Based on Medicare’s Minimum Eligibility Thresholds eTable 1. Additional Characteristics of MTM-Eligible Beneficiaries and Completers vs Noncompleters of CMRs Before vs After 2016 Star Rating Measure eTable 2. Characteristics of MTM-Eligible Beneficiaries and Users vs Nonusers of CMRs Before vs After 2016 Star Rating Quality Measure (Using the Simulated MTM-Eligible Cohort Based on Medicare’s Minimum Eligibility Thresholds) eTable 3. Patient Characteristics Associated With MTM CMR Completion After Adjusting for Time Trends (n=561,950) eTable 4. Descriptive Trends of Cohort Simulated as Eligible for MTM Based on CMS Minimum Thresholds and Breakdown of Cohort Based on Inclusion Criteria eTable 5. Change in MTM CMR Completion Rates Before vs After 2016 Star Rating Measure Using the Simulated MTM-Eligible Cohort Based on Medicare’s Minimum Eligibility Thresholds (n=663,315) eTable 6. Model-based Estimated Probability of MTM CMR Completion by Subgroup and Year [file jamahealthforum-e240807-s001.pdf]

Supplemental Online Content

Hung A, Wilson L, Smith VA, et al. Comprehensive Medication review completion rates and disparities after medicare star rating measure. *JAMA Health Forum*. Published online May 3, 2024. doi:10.1001/jamahealthforum.2024.0807

eAppendix. Additional Background on Star Rating Measures
eFigure 1. Cohort Inclusion Diagram
eMethods. Simulated Cohort Definition Based on Medicare's Minimum Eligibility Thresholds
eTable 1. Additional Characteristics of MTM-Eligible Beneficiaries and Completers vs Noncompleters of CMRs Before vs After 2016 Star Rating Measure
eTable 2. Characteristics of MTM-Eligible Beneficiaries and Users vs Nonusers of CMRs Before vs After 2016 Star Rating Quality Measure (Using the Simulated MTM-Eligible Cohort Based on Medicare's Minimum Eligibility Thresholds)
eTable 3. Patient Characteristics Associated With MTM CMR Completion After Adjusting for Time Trends (n=561,950)
eTable 4. Descriptive Trends of Cohort Simulated as Eligible for MTM Based on CMS Minimum Thresholds and Breakdown of Cohort Based on Inclusion Criteria
eTable 5. Change in MTM CMR Completion Rates Before vs After 2016 Star Rating Measure Using the Simulated MTM-Eligible Cohort Based on Medicare's Minimum Eligibility Thresholds (n=663,315)
eTable 6. Model-based Estimated Probability of MTM CMR Completion by Subgroup and Year

This supplemental material has been provided by the authors to give readers additional information about their work.

© 2024 Hung A et al. *JAMA Health Forum.*

eAppendix. Additional Background on Star Rating measures

From 2013 to 2020, the number of Star Rating quality measures has fluctuated between 13 and 18 each year. Common measures include:

- Medication Adherence for Diabetes Medications
- Medication Adherence for Hypertension (RAS antagonist)
- Medication Adherence for Cholesterol (Statins)
- Statin Use in Persons with Diabetes
- Diabetes Treatment
- High Risk Medication
- Medicare Plan Finder Price Accuracy
- Rating of Drug Plan
- Complaints about the Drug Plan
- Enrollment Timeliness
- Appeals Auto-Forward
- Appeals Upheld
- Beneficiary Access and Performance Problems
- Drug Plan Quality Improvement
- Getting Needed Prescription Dugs
- Call Center – Foreign Language Interpreter and TTY Availability
- MTM Program Completion Rate for CMR

Each measure included in the set of Star Rating quality measures is assigned a star rating, ranging from 1 to 5 (with 5 being the highest). Each of these individual-measure star ratings are aggregated to determine an overall star rating for each Part D plan. How much each measure's star rating contributes to the overall rating depends on the weight assigned to the individual measure.[1]

When beneficiaries are shopping for a Part D plan during open enrollment, they can see the overall star rating and each individual measure star rating for each plan they are considering. This creates an incentive for Part D plans to care about and improve their star ratings, in order to increase enrollment numbers. Furthermore, Star Ratings are associated with financial incentives (such as quality bonus payments and the portion of the difference between the benchmark and the plan's bid that is paid to the plan) for Medicare Advantage plans. These financial incentives can be lucrative with an estimated $12.8 billion in bonus payments paid to Medicare Advantage plans in 2023.[2]

References:

1) PQA Measure Use in CMS' Part D Quality Programs. Pharmacy Quality Alliance. Apr 2023. Available from: https://www.pqaalliance.org/medicare-part-d.
2) Biniek JF, Damico A, and Neuman T. Spending on Medicare Advantage Quality Bonus Payments Will Reach at Least $12.8 Billion in 2023. Aug 9, 2023. Available from: https://www.kff.org/medicare/issue-brief/spending-on-medicare-advantage-quality-bonus-payments-will-reach-at-least-12-8-billion-in-2023/.

© 2024 Hung A et al. *JAMA Health Forum.*

eFigure 1. Cohort Inclusion Diagram

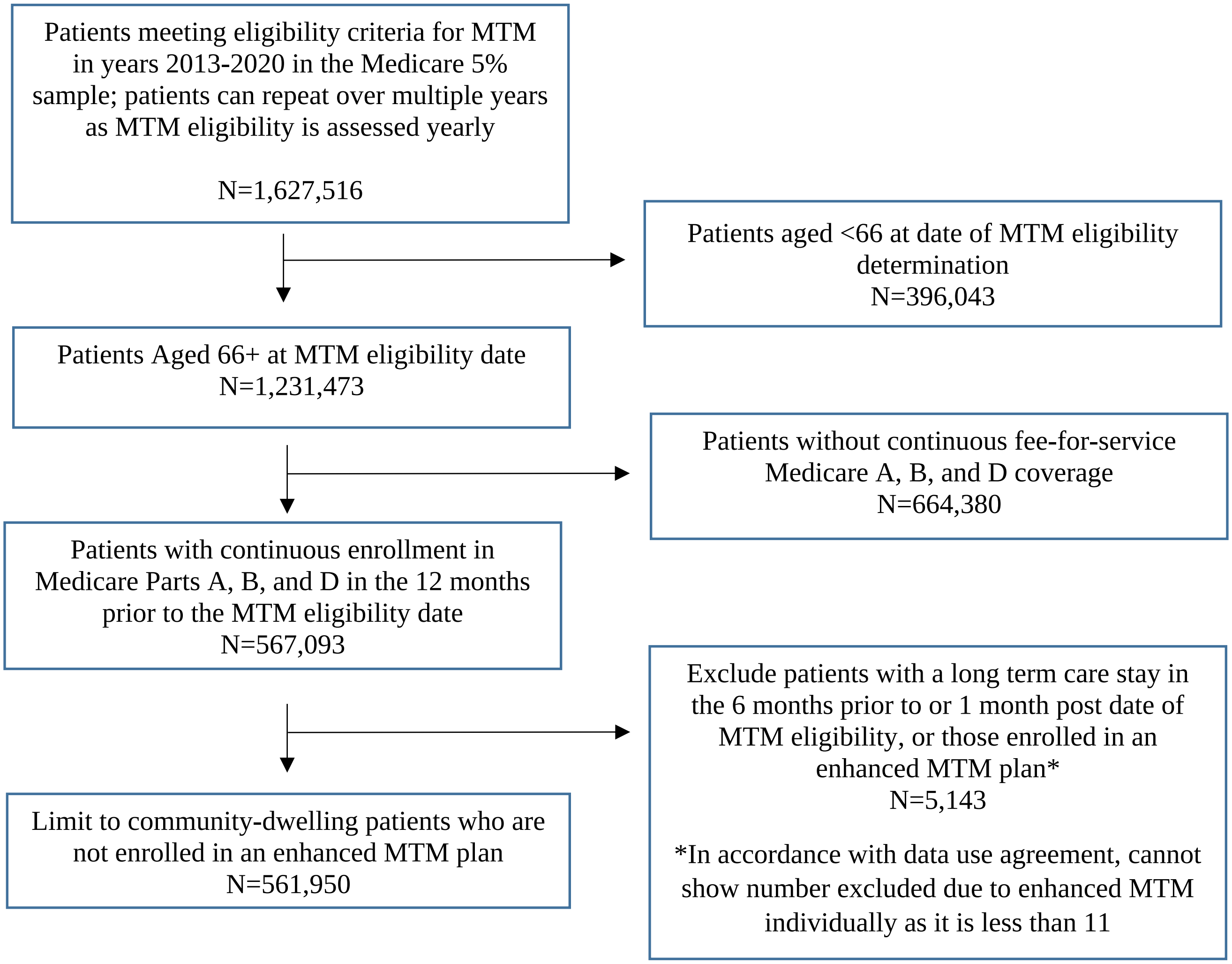

MTM = medication therapy management

eMethods. Simulated Cohort Definition Based on Medicare's Minimum Eligibility Thresholds

- 3 or more of the core chronic conditions in the calendar year of interest: Diabetes, Congestive Heart Failure, Hyperlipidemia, Alzheimer's Disease, End-stage Renal Disease, Hypertension, Chronic Obstructive Pulmonary Disease, Bone Disease-Arthritis (i.e., Osteoarthritis, Rheumatoid Arthritis), Mental Health Disorders (i.e., Psychoses, Depression);
- 8 or more unique prescription drugs in the calendar year; and
- Total Part D drug costs of $4255 or more in the calendar year

© 2024 Hung A et al. *JAMA Health Forum.*

eTable 1. Additional Characteristics of MTM-Eligible Beneficiaries and Completers vs Noncompleters of CMRs Before vs After 2016 Star Rating Measure

	Before Star Rating Quality Measure (2013-2015)				After Star Rating Quality Measure (2016-2020)				SMD comparing MTM-eligible before vs after quality measure
	Overall MTM-eligible (n=253,561)	No CMR (n=222,134)	CMR (n=31,427)	SMD comparing no CMR vs CMR	Overall MTM-eligible (n=308,389)	No CMR (n=223,859)	CMR (n=84,530)	SMD comparing no CMR vs CMR	
Comorbid Conditions									
Diabetes	115,067 (45.4%)	101,813 (45.8%)	13,254 (42.2%)	7.4%	134,882 (43.7%)	101,398 (45.3%)	33,484 (39.6%)	11.5%	3.3%
Heart failure	41,081 (16.2%)	36,731 (16.5%)	4,350 (13.8%)	7.5%	52,971 (17.2%)	40,052 (17.9%)	12,919 (15.3%)	7.0%	2.6%
Hyperlipidemia	190,257 (75.0%)	166,430 (74.9%)	23,827 (75.8%)	2.1%	242,223 (78.5%)	175,765 (78.5%)	66,458 (78.6%)	0.3%	8.3%
Hypertension	150,855 (59.5%)	133,353 (60.0%)	17,502 (55.7%)	8.8%	175,181 (56.8%)	130,396 (58.2%)	44,785 (53.0%)	10.6%	5.5%
Chronic obstructive pulmonary disease	56,929 (22.5%)	49,759 (22.4%)	7,170 (22.8%)	1.0%	74,947 (24.3%)	54,665 (24.4%)	20,282 (24.0%)	1.0%	4.4%
Asthma	29,088 (11.5%)	25,069 (11.3%)	4,019 (12.8%)	4.6%	39,925 (12.9%)	27,992 (12.5%)	11,933 (14.1%)	4.7%	4.5%
Osteoporosis	44,438 (17.5%)	39,361 (17.7%)	5,077 (16.2%)	4.2%	55,881 (18.1%)	41,826 (18.7%)	14,055 (16.6%)	5.4%	1.6%
Depression	33,391 (13.2%)	29,743 (13.4%)	3,648 (11.6%)	5.4%	43,920 (14.2%)	33,282 (14.9%)	10,638 (12.6%)	6.6%	3.1%
Hearing loss	5,427 (2.1%)	4,760 (2.1%)	667 (2.1%)	0.1%	14,023 (4.5%)	10,167 (4.5%)	3,856 (4.6%)	0.1%	13.4%
Dementia	32,055 (12.6%)	29,778 (13.4%)	2,277 (7.2%)	20.3%	32,688 (10.6%)	25,930 (11.6%)	6,758 (8.0%)	12.1%	6.4%
Chronic kidney disease	38,401 (15.1%)	34,157 (15.4%)	4,244 (13.5%)	5.3%	55,811 (18.1%)	42,073 (18.8%)	13,738 (16.3%)	6.7%	7.9%
Utilization in 12 months prior to eligibility									
Any inpatient stay	83,775 (33.0%)	74,175 (33.4%)	9,600 (30.5%)	6.1%	103,143 (33.4%)	75,717 (33.8%)	27,426 (32.4%)	2.9%	0.9%
Any emergency room visit	120,488 (47.5%)	106,162 (47.8%)	14,326 (45.6%)	4.4%	152,876 (49.6%)	111,335 (49.7%)	41,541 (49.1%)	1.2%	4.1%

© 2024 Hung A et al. *JAMA Health Forum.*

Outpatient visit count, Median (Q1, Q3)	5.0 (2.0, 10.0)	5.0 (2.0, 10.0)	5.0 (2.0, 11.0)	5.5%	5.0 (2.0, 11.0)	5.0 (2.0, 11.0)	6.0 (2.0, 12.0)	5.3%	7.8%
Prior year costs to Medicare, Median (Q1, Q3)	15314.4 (8091.1, 33135.8)	15373.5 (8076.8, 33582.3)	14957.0 (8193.2, 30481.8)	7.6%	18075.2 (9492.4, 37607.7)	17952.8 (9384.9, 37877.5)	18379.6 (9774.3, 36918.1)	1.2%	9.9%
Number of unique drugs filled in prior year, Median (Q1, Q3)	15.0 (12.0, 20.0)	15.0 (12.0, 20.0)	15.0 (12.0, 20.0)	1.2%	16.0 (13.0, 20.0)	16.0 (13.0, 20.0)	16.0 (13.0, 21.0)	2.1%	9.5%
Number of prescription fills in prior year, Median (Q1, Q3)	65.0 (43.0, 99.0)	65.0 (43.0, 100.0)	61.0 (42.0, 93.0)	10.6%	62.0 (44.0, 92.0)	62.0 (44.0, 93.0)	61.0 (44.0, 89.0)	6.0%	3.4%
Prior year out-of-pocket medication costs, Median (Q1, Q3)	717.4 (163.9, 1609.8)	701.7 (158.0, 1604.7)	815.6 (217.6, 1646.0)	5.3%	824.9 (150.8, 1745.1)	821.5 (143.8, 1760.8)	833.3 (171.7, 1702.5)	0.3%	5.6%
Number of unique PIMs filled in prior year, Median (Q1, Q3)	1.0 (0.0, 1.0)	1.0 (0.0, 1.0)	1.0 (0.0, 1.0)	0.5%	0.0 (0.0, 1.0)	0.0 (0.0, 1.0)	0.0 (0.0, 1.0)	0.6%	17.1%
Number of PIM prescription fills in prior year, Median (Q1, Q3)	1.0 (0.0, 5.0)	1.0 (0.0, 5.0)	1.0 (0.0, 5.0)	2.4%	0.0 (0.0, 4.0)	0.0 (0.0, 4.0)	0.0 (0.0, 4.0)	2.2%	17.6%

CMR = comprehensive medication review; MTM = medication therapy management; PIM = potentially inappropriate medication; Q = quarter; SMD = standardized mean difference

© 2024 Hung A et al. *JAMA Health Forum.*

eTable 2. Characteristics of MTM-Eligible Beneficiaries and Users vs Nonusers of CMRs Before vs After 2016 Star Rating Quality Measure (Using the Simulated MTM-Eligible Cohort Based on Medicare's Minimum Eligibility Thresholds)

	**Before Star Rating Quality Measure (2013-2015)**				**After Star Rating Quality Measure (2016-2020)**				**SMD comparing MTM-eligible before vs after quality measure**
	**Overall MTM-eligible (n=**225,044**)**	**No CMR (n=**211,500**)**	**CMR (n=**13,544**)**	**SMD comparing no CMR vs CMR**	**Overall MTM-eligible (n=**438,271**)**	**No CMR (n=**389,622**)**	**CMR (n=**48,649**)**	**SMD comparing no CMR vs CMR**	
Age at date of MTM eligibility, Median (Q1, Q3)	76.0 (71.0, 82.0)	76.0 (71.0, 82.0)	75.0 (70.0, 80.0)	17.0%	75.0 (71.0, 81.0)	75.0 (71.0, 81.0)	75.0 (71.0, 80.0)	11.1%	4.2%
Male sex	78,044 (34.7%)	73,491 (34.7%)	4,553 (33.6%)	2.4%	180,118 (41.1%)	160,047 (41.1%)	20,071 (41.3%)	0.4%	13.3%
Race/Ethnicity				22.8%				10.0%	13.2%
Unknown	1,231 (0.5%)	1,176 (0.6%)	55 (0.4%)		7,023 (1.6%)	6,259 (1.6%)	764 (1.6%)		
White	176,907 (78.6%)	165,552 (78.3%)	11,355 (83.8%)		353,547 (80.7%)	313,416 (80.4%)	40,131 (82.5%)		
Black	23,703 (10.5%)	22,217 (10.5%)	1,486 (11.0%)		37,642 (8.6%)	33,180 (8.5%)	4,462 (9.2%)		
Other	4,145 (1.8%)	4,011 (1.9%)	134 (1.0%)		8,168 (1.9%)	7,457 (1.9%)	711 (1.5%)		
Asian	9,596 (4.3%)	9,380 (4.4%)	216 (1.6%)		17,051 (3.9%)	15,687 (4.0%)	1,364 (2.8%)		
Hispanic	8,245 (3.7%)	7,997 (3.8%)	248 (1.8%)		12,316 (2.8%)	11,303 (2.9%)	1,013 (2.1%)		
North American Native	1,217 (0.5%)	1,167 (0.6%)	50 (0.4%)		2,524 (0.6%)	2,320 (0.6%)	204 (0.4%)		
Dual-Medicaid Enrollment/LIS				16.2%				4.3%	27.8%
No dual-Medicaid enrollment and no LIS	107,441 (47.7%)	99,976 (47.3%)	7,465 (55.1%)		269,364 (61.5%)	238,740 (61.3%)	30,624 (62.9%)		
LIS only	13,480 (6.0%)	12,676 (6.0%)	804 (5.9%)		18,915 (4.3%)	16,681 (4.3%)	2,234 (4.6%)		
Dual-Medicaid enrollment	104,123 (46.3%)	98,848 (46.7%)	5,275 (38.9%)		149,992 (34.2%)	134,201 (34.4%)	15,791 (32.5%)		
Geographic Region				17.1%				6.9%	7.5%

© 2024 Hung A et al. *JAMA Health Forum.*

Northeast	55,043 (24.5%)	52,064 (24.6%)	2,979 (22.0%)		99,276 (22.7%)	88,471 (22.7%)	10,805 (22.2%)		
Midwest	43,272 (19.2%)	40,109 (19.0%)	3,163 (23.4%)		86,324 (19.7%)	75,609 (19.4%)	10,715 (22.0%)		
Other	183 (0.1%)	>170	*		458 (0.1%)	431 (0.1%)	27 (0.1%)		
South	89,813 (39.9%)	84,042 (39.7%)	5,771 (42.6%)		169,479 (38.7%)	151,513 (38.9%)	17,966 (36.9%)		
West	36,733 (16.3%)	35,110 (16.6%)	1,623 (12.0%)		82,734 (18.9%)	73,598 (18.9%)	9,136 (18.8%)		
Rural residence	57,381 (25.5%)	53,297 (25.2%)	4,084 (30.2%)	11.1%	107,640 (24.6%)	94,383 (24.2%)	13,257 (27.3%)	6.9%	2.2%
Charlson Comorbidity Score, Median (Q1, Q3)	4.0 (2.0, 6.0)	4.0 (2.0, 6.0)	4.0 (3.0, 6.0)	15.4%	4.0 (3.0, 6.0)	4.0 (2.0, 6.0)	5.0 (3.0, 7.0)	24.7%	12.0%
Comorbid Conditions									
Diabetes	146,036 (64.9%)	135,451 (64.0%)	10,585 (78.2%)	31.5%	280,736 (64.1%)	242,038 (62.1%)	38,698 (79.5%)	39.1%	1.7%
Heart failure	71,307 (31.7%)	66,492 (31.4%)	4,815 (35.6%)	8.7%	142,134 (32.4%)	123,351 (31.7%)	18,783 (38.6%)	14.6%	1.6%
Hyperlipidemia	172,057 (76.5%)	161,050 (76.1%)	11,007 (81.3%)	12.5%	346,089 (79.0%)	305,482 (78.4%)	40,607 (83.5%)	12.9%	6.0%
Hypertension	215,481 (95.8%)	202,294 (95.6%)	13,187 (97.4%)	9.4%	419,277 (95.7%)	371,776 (95.4%)	47,501 (97.6%)	12.2%	0.4%
Chronic obstructive pulmonary disease	108,374 (48.2%)	101,064 (47.8%)	7,310 (54.0%)	12.4%	206,576 (47.1%)	180,297 (46.3%)	26,279 (54.0%)	15.5%	2.0%
Asthma	28,230 (12.5%)	26,218 (12.4%)	2,012 (14.9%)	7.2%	52,366 (11.9%)	45,204 (11.6%)	7,162 (14.7%)	9.2%	1.8%
Osteoporosis	108,491 (48.2%)	102,251 (48.3%)	6,240 (46.1%)	4.6%	211,960 (48.4%)	188,863 (48.5%)	23,097 (47.5%)	2.0%	0.3%
Depression	65,908 (29.3%)	61,746 (29.2%)	4,162 (30.7%)	3.4%	130,891 (29.9%)	114,994 (29.5%)	15,897 (32.7%)	6.8%	1.3%
Hearing loss	5,202 (2.3%)	4,925 (2.3%)	277 (2.0%)	1.9%	19,826 (4.5%)	17,637 (4.5%)	2,189 (4.5%)	0.1%	12.2%
Dementia	22,482 (10.0%)	21,659 (10.2%)	823 (6.1%)	15.3%	32,582 (7.4%)	29,790 (7.6%)	2,792 (5.7%)	7.6%	9.1%
Chronic kidney disease	71,228 (31.7%)	66,525 (31.5%)	4,703 (34.7%)	7.0%	157,248 (35.9%)	137,397 (35.3%)	19,851 (40.8%)	11.4%	9.0%
Utilization in 12 months prior to eligibility									

© 2024 Hung A et al. *JAMA Health Forum.*

Any inpatient stay	67,801 (30.1%)	63,614 (30.1%)	4,187 (30.9%)	1.8%	123,827 (28.3%)	108,839 (27.9%)	14,988 (30.8%)	6.3%	4.1%
Any emergency room visit	103,594 (46.0%)	97,157 (45.9%)	6,437 (47.5%)	3.2%	199,729 (45.6%)	175,995 (45.2%)	23,734 (48.8%)	7.2%	0.9%
Outpatient visit count, Median (Q1, Q3)	4.0 (1.0, 10.0)	4.0 (1.0, 10.0)	5.0 (2.0, 11.0)	12.4%	5.0 (2.0, 11.0)	5.0 (2.0, 10.0)	6.0 (2.0, 12.0)	11.4%	4.8%
Prior year costs to Medicare, Median (Q1, Q3)	17353.7 (10112.1, 34593.0)	17254.6 (10037.2, 34541.7)	18987.6 (11416.3, 35317.0)	0.6%	17550.5 (9885.1, 35659.3)	17184.4 (9690.8, 35187.5)	20571.2 (11748.1, 39346.1)	6.9%	3.7%
Number of unique drugs filled in prior year, Median (Q1, Q3)	16.0 (12.0, 20.0)	16.0 (12.0, 20.0)	18.0 (14.0, 22.0)	27.9%	15.0 (11.0, 19.0)	15.0 (11.0, 19.0)	17.0 (13.0, 22.0)	38.6%	13.5%
Number of prescription fills in prior year, Median (Q1, Q3)	65.0 (43.0, 97.0)	65.0 (43.0, 97.0)	73.0 (50.0, 106.0)	18.3%	55.0 (38.0, 82.0)	54.0 (37.0, 80.0)	65.0 (47.0, 93.0)	27.9%	22.4%
Prior year out-of-pocket medication costs, Median (Q1, Q3)	338.4 (127.7, 1479.7)	326.4 (125.3, 1454.4)	667.6 (165.6, 1807.7)	12.9%	668.8 (127.1, 1869.7)	654.1 (125.1, 1860.2)	782.4 (146.2, 1946.0)	1.9%	11.7%
Number of unique PIMs filled in prior year, Median (Q1, Q3)	1.0 (0.0, 1.0)	1.0 (0.0, 1.0)	1.0 (0.0, 1.0)	7.3%	0.0 (0.0, 1.0)	0.0 (0.0, 1.0)	0.0 (0.0, 1.0)	9.2%	25.5%
Number of PIM prescription fills in prior year, Median (Q1, Q3)	1.0 (0.0, 5.0)	1.0 (0.0, 5.0)	1.0 (0.0, 6.0)	7.2%	0.0 (0.0, 3.0)	0.0 (0.0, 3.0)	0.0 (0.0, 4.0)	9.1%	25.8%

CMR = comprehensive medication review; LIS = low-income subsidy; MTM = medication therapy management; PIM = potentially inappropriate medication; Q = quarter; SMD = standardized mean difference

© 2024 Hung A et al. *JAMA Health Forum.*

eTable 3. Patient Characteristics Associated With MTM CMR Completion After Adjusting for Time Trends (n=561,950)

	Adjusted Risk Ratio (95% Confidence Interval)
PATIENT CHARACTERISTICS	
Age (continuous in one-year increments)	0.99 (0.99-0.99)
Male	0.94 (0.92-0.95)
Race or ethnicity (ref. White)	
Unknown	0.84 (0.78-0.91)
Black	1.04 (1.01-1.07)*
Other	0.78 (0.72-0.84)
Asian	0.72 (0.68-0.76)
Hispanic	0.66 (0.62-0.70)
North American Native	0.57 (0.50-0.64)
Dual-Medicaid Enrollment/LIS (ref. None)	
LIS only	0.96 (0.92-1.01)
Dual-Medicaid enrollment	0.87 (0.85-0.89)
Region of residence (ref. Northeast)	
Midwest	0.90 (0.87-0.92)
Other	0.74 (0.57-0.95)
South	0.88 (0.86-0.90)
West	0.93 (0.91-0.96)
Rural residence (ref. No)	1.12 (1.10-1.14)
Charlson Comorbidity Score (continuous in one-unit increments)	0.95 (0.95-0.95)
Had inpatient stay in prior year	0.98 (0.96-0.99)*
Had emergency room visit in prior year	0.98 (0.97-1.00)
Number of outpatient visits in prior year	1.01 (1.00-1.01)
Inflation-adjusted costs in prior year	1.00 (1.00-1.00)
Number of unique medications in prior year	1.01 (1.01-1.02)
Number of prescription fills in prior year	1.00 (1.00-1.00)
Inflation-adjusted out-of-pocket medication costs in prior year	1.00 (1.00-1.00)
Number of unique PIMs in prior year	1.00 (0.99-1.00)
TIME TERMS	
Slope 2013-2015	1.30 (1.28-1.31)
Change in intercept at 2016	1.07 (1.04-1.09)
Slope 2016-2020	1.26 (1.22-1.30)

CMR = comprehensive medication review; LIS = low-income subsidy; MTM = medication therapy management; PIM = potentially inappropriate medication
*Confidence interval included 1.00 in sensitivity analyses replacing the comorbidity score with individual comorbidities

eTable 4. Descriptive Trends of Cohort Simulated as Eligible for MTM Based on CMS Minimum Thresholds and Breakdown of Cohort Based on Inclusion Criteria

Year	Enrolled in Part D 12 months prior and 12 months following start of calendar year (N=7,166,092)	Criterion #1: 3+ chronic conditions (N=3,738,255)	Criterion #2: Part D costs of $4255+ in calendar year (N=1,103,403)	Criterion #3: 8+ prescription drugs in calendar year (N=1,029,665)	Final Simulated Cohort: Meet all 3 criteria and after exclusions for long-term care and enhanced MTM plans (N=662,332)
2013	813,085	424,619	128,828	122,534	71,243
2014	892,303	464,379	139,898	132,159	78,693
2015	849,187	442,452	130,934	123,312	74,678
2016	907,705	473,815	135,189	126,766	79,054
2017	914,690	483,256	138,487	129,625	83,146
2018	920,816	488,937	142,264	132,261	88,307
2019	917,352	489,591	144,075	133,182	93,105
2020	950,954	471,206	143,728	129,826	94,106

Each column represents a subset of the column to the left

© 2024 Hung A et al. *JAMA Health Forum.*

eTable 5. Change in MTM CMR Completion Rates Before vs After 2016 Star Rating Measure Using the Simulated MTM-Eligible Cohort Based on Medicare's Minimum Eligibility Thresholds (n=663,315)

	Adjusted Risk Ratio (95% Confidence Interval)	P-value*
Model 1 (averaged over all populations)		
Slope 2013-2015	1.35 (1.32-1.38)	n/a
Change in intercept at 2016	1.05 (1.01-1.08)	n/a
Slope 2016-2020	1.13 (1.08-1.19)	n/a
Model 2 (by racial and ethnic subgroup)		
Race and ethnicity		
White beneficiaries (ref group)		
Slope 2013-2015	1.34 (1.30-1.37)	n/a
Change in intercept at 2016	1.06 (1.02-1.10)	n/a
Slope 2016-2020	1.12 (1.06-1.18)	n/a
Black beneficiaries		
Slope 2013-2015	1.31 (1.18-1.45)	0.551
Change in intercept at 2016	0.94 (0.81-1.10)	0.044
Slope 2016-2020	1.18 (0.96-1.46)	<.0001
Hispanic beneficiaries		
Slope 2013-2015	1.49 (1.22-1.81)	0.205
Change in intercept at 2016	1.03 (0.77-1.37)	0.837
Slope 2016-2020	1.25 (0.83-1.87)	<.0001
Asian beneficiaries		
Slope 2013-2015	1.62 (1.32-2.00)	0.030
Change in intercept at 2016	1.16 (0.88-1.53)	0.419
Slope 2016-2020	1.25 (0.82-1.92)	<.0001
North American Native beneficiaries		
Slope 2013-2015	1.44 (0.99-2.11)	0.669
Change in intercept at 2016	1.11 (0.62-1.99)	0.840
Slope 2016-2020	1.08 (0.49-2.38)	0.512
Unknown race		
Slope 2013-2015	1.47 (1.02-2.11)	0.580
Change in intercept at 2016	1.11 (0.71-1.74)	0.795
Slope 2016-2020	1.20 (0.58-2.50)	0.021
Other race		
Slope 2013-2015	1.43 (1.13-1.82)	0.527
Change in intercept at 2016	1.30 (0.93-1.82)	0.163
Slope 2016-2020	1.15 (0.70-1.87)	0.418
Model 3 (by socioeconomic subgroup)		
Lower income		
Neither Dual-Medicaid nor LIS enrollee (ref group)		
Slope 2013-2015	1.36 (1.31-1.40)	n/a
Change in intercept at 2016	1.11 (1.06-1.16)	n/a
Slope 2016-2020	1.09 (1.01-1.17)	n/a
Dual-Medicaid enrollee		
Slope 2013-2015	1.33 (1.23-1.45)	0.468
Change in intercept at 2016	0.93 (0.83-1.05)	<.0001
Slope 2016-2020	1.22 (1.03-1.45)	<.0001
LIS-only enrollee		
Slope 2013-2015	1.25 (1.10-1.44)	0.118
Change in intercept at 2016	0.96 (0.78-1.18)	0.066
Slope 2016-2020	1.19 (0.90-1.57)	<.0001

CMR = comprehensive medication review; LIS = low-income subsidy; MTM = medication therapy management
*Test between subgroup and reference group for a given time term
**All three models adjusted for age, sex, race, ethnicity, dual-Medicaid enrollment/low-income subsidy, region, rural residence, Charlson Comorbidity Score, and prior-year utilization (e.g., had inpatient stay, had emergency room visit, number of outpatient visits, inflation-adjusted costs, number of unique medications, number prescription fills, inflation-adjusted out-of-pocket medication costs, number of potentially inappropriate medications). Models 2 and 3 included interaction terms among each of the time terms and each of the racial/ethnic and socioeconomic subgroups, respectively.

© 2024 Hung A et al. *JAMA Health Forum.*

eTable 6. Model-based Estimated Probability of MTM CMR Completion by Subgroup and Year

	Adjusted Probability (95% Confidence Interval)	P-value*
Model 2 (by racial and ethnic subgroup)		
White beneficiaries (reference group)		
2013	0.10 (0.09-0.10)	n/a
2014	0.12 (0.11-0.13)	n/a
2015	0.15 (0.14-0.16)	n/a
2016	0.19 (0.18-0.20)	n/a
2017	0.23 (0.22-0.24)	n/a
2018	0.27 (0.26-0.28)	n/a
2019	0.32 (0.31-0.33)	n/a
2020	0.37 (0.36-0.38)	n/a
Black beneficiaries		
2013	0.10 (0.09-0.11)	0.096
2014	0.12 (0.12-0.13)	0.096
2015	0.16 (0.15-0.17)	0.096
2016	0.19 (0.17-0.21)	0.939
2017	0.23 (0.21-0.25)	0.939
2018	0.27 (0.25-0.30)	0.939
2019	0.32 (0.29-0.35)	0.939
2020	0.37 (0.34-0.40)	0.939
Hispanic beneficiaries		
2013	0.05 (0.04-0.06)	<.001
2014	0.06 (0.05-0.07)	<.001
2015	0.08 (0.07-0.09)	<.001
2016	0.13 (0.10-0.17)	<.001
2017	0.16 (0.13-0.20)	<.001
2018	0.19 (0.15-0.24)	<.001
2019	0.23 (0.19-0.28)	<.001
2020	0.27 (0.22-0.33)	<.001
Asian beneficiaries		
2013	0.04 (0.03-0.05)	<.001
2014	0.06 (0.06-0.07)	<.001
2015	0.08 (0.07-0.09)	<.001
2016	0.10 (0.08-0.13)	<.001
2017	0.13 (0.10-0.16)	<.001
2018	0.15 (0.12-0.19)	<.001
2019	0.19 (0.15-0.23)	<.001
2020	0.22 (0.18-0.27)	<.001
North American Native beneficiaries		
2013	0.07 (0.05-0.11)	0.117
2014	0.08 (0.06-0.10)	0.117
2015	0.10 (0.08-0.13)	0.117
2016	0.15 (0.09-0.23)	0.208
2017	0.18 (0.11-0.27)	0.208
2018	0.21 (0.14-0.32)	0.208
2019	0.25 (0.16-0.37)	0.208
2020	0.30 (0.20-0.42)	0.208
Unknown race		
2013	0.06 (0.04-0.09)	0.004
2014	0.08 (0.06-0.10)	0.004
2015	0.10 (0.08-0.12)	0.004
2016	0.14 (0.09-0.21)	0.004
2017	0.17 (0.11-0.25)	0.004
2018	0.20 (0.14-0.29)	0.004
2019	0.24 (0.16-0.34)	0.004
2020	0.29 (0.20-0.40)	0.004

© 2024 Hung A et al. *JAMA Health Forum.*

Other race		
2013	0.07 (0.05-0.08)	<0.001
2014	0.08 (0.07-0.10)	<0.001
2015	0.11 (0.09-0.12)	<0.001
2016	0.16 (0.12-0.21)	0.147
2017	0.19 (0.15-0.25)	0.147
2018	0.23 (0.18-0.29)	0.147
2019	0.27 (0.21-0.34)	0.147
2020	0.32 (0.25-0.39)	0.147
Model 3 (by socioeconomic subgroup)		
Neither dual-Medicaid enrollee nor LIS enrollee (reference group)		
2013	0.10 (0.09-0.10)	n/a
2014	0.12 (0.12-0.13)	n/a
2015	0.16 (0.15-0.16)	n/a
2016	0.20 (0.19-0.21)	n/a
2017	0.23 (0.22-0.24)	n/a
2018	0.27 (0.26-0.28)	n/a
2019	0.31 (0.30-0.33)	n/a
2020	0.36 (0.35-0.37)	n/a
Dual-Medicaid enrollee		
2013	0.08 (0.08-0.08)	<0.001
2014	0.10 (0.09-0.10)	<0.001
2015	0.12 (0.12-0.13)	<0.001
2016	0.18 (0.16-0.19)	<0.001
2017	0.21 (0.19-0.22)	<0.001
2018	0.24 (0.23-0.26)	<0.001
2019	0.28 (0.27-0.30)	<0.001
2020	0.33 (0.31-0.35)	<0.001
LIS-only enrollee		
2013	0.10 (0.09-0.11)	0.663
2014	0.12 (0.11-0.12)	0.663
2015	0.15 (0.14-0.16)	0.663
2016	0.21 (0.19-0.24)	0.098
2017	0.25 (0.22-0.28)	0.098
2018	0.29 (0.26-0.33)	0.098
2019	0.34 (0.30-0.37)	0.098
2020	0.38 (0.34-0.42)	0.098

CMR = comprehensive medication review; LIS = low-income subsidy; MTM = medication therapy management

*Test between subgroup and reference group for a given year

**The subgroup model (Model 2) adjusted for age, sex, race, ethnicity, dual-Medicaid enrollment/low-income subsidy, region, rural residence, Charlson Comorbidity Score, and prior-year utilization (e.g., had inpatient stay, had emergency room visit, number of outpatient visits, inflation-adjusted costs, number of unique medications, number prescription fills, inflation-adjusted out-of-pocket medication costs, number of potentially inappropriate medications)

© 2024 Hung A et al. *JAMA Health Forum.*
