# Supplementary material for: Comprehensive Medication Review Completion Rates and Disparities After Medicare Star Rating Measure
Source: JAMA Health Forum. 2024 May 3;5(5):e240807. doi: 10.1001/jamahealthforum.2024.0807 (PMC11069085; doi:10.1001/jamahealthforum.2024.0807)
Supplement: Supplement 2. — Data Sharing Statement [file jamahealthforum-e240807-s002.pdf]

Data Sharing Statement

Hung. Comprehensive Medication Review Completion Rates and Disparities After Medicare Star Rating Measure. *JAMA Health Forum*. Published May 03, 2024.
doi:10.1001/jamahealthforum.2024.0807

Data
Data available: No

Additional Information
Explanation for why data not available: This study was conducted using Medicare data via a data use agreement with the Centers for Medicare & Medicaid Services. Access to patient-level data is not allowed via the data use agreement.
